# Der Diagnostik-Parcours: Eine neue Lernform im Studierendenkurs der Hals-Nasen-Ohren-Heilkunde

**DOI:** 10.1007/s00106-020-00929-7

**Published:** 2020-09-03

**Authors:** V. Vielsmeier, C. Bohr, N. Müller

**Affiliations:** Klinik und Poliklinik für Hals-Nasen-Ohrenheilkunde, Universitätsklinikum Regensburg, Franz-Josef-Strauß-Allee 11, 93053 Regensburg, Deutschland

**Keywords:** Medizinische Lehre, Studierendenzahlen, Diagnostische Untersuchungen, Sinne, Evaluation, Medical education, Number of students, Diagnostics, Senses, Evaluation

## Abstract

**Hintergrund:**

Fehlende praktische Übungen sowie Anstieg der Studierendenzahlen führen zu der Notwendigkeit innovativer Lehrmethoden. Wir etablierten einen Diagnostik-Parcours zur Darstellung der Sinne unseres Faches.

**Ziel der Arbeit:**

Das vorwiegende Ziel der Einführung des Diagnostik-Parcours war die kompakte Darstellung der Funktionsdiagnostik einer HNO(Hals-Nasen-Ohrenheilkunde)-Abteilung sowie die praktische und abwechslungsreiche Gestaltung des bestehenden Blockpraktikums.

**Material und Methoden:**

Am ersten Tag des fünftägigen Blockpraktikums wird ein „HNO-Spiegelkurs“ durchgeführt, bei dem die Studierenden lernen, einen HNO-Spiegelbefund zu erheben. Nach der Einteilung auf den HNO-Stationen, in der Poliklinik sowie in den Operationssälen erfolgt am 5. Tag der Diagnostik-Parcours, bei dem vier verschiedene Stationen der Diagnostik einer HNO-Klinik in praktischen Übungen durchlaufen werden.

**Ergebnisse:**

In der Evaluation des Blockpraktikums konnten wir unsere Bewertung durch Einführung des Diagnostik-Parcours signifikant verbessern. So lag im Sommersemester 2019 die Note bei 1,4 bei 38 % von *n* = 105. Auch persönliche Rückmeldungen sowie mehrere Famulaturanfragen verdeutlichen das positive Feedback.

**Schlussfolgerung:**

Der Vorteil des Diagnostik-Parcours ist die Möglichkeit, praktische Fertigkeiten direkt anzuwenden. Außerdem ist die kleine Gruppengröße von maximal fünf Studierenden als positiv zu werten. Somit kann das Fach der Hals-Nasen-Ohren-Heilkunde mit seinem abwechslungsreichen Charakter attraktiv dargestellt werden. Auch wenn der vorgestellte Parcours einen hohen personellen Aufwand bedeutet, sollte dies in der universitären Lehre ermöglicht werden.

## Abstract (en)

**Background:**

A lack of practical courses and increasing numbers of students generate a requirement for innovative teaching methods. We have established a diagnostic course representing the essence of our discipline of otorhinolaryngology.

**Objective:**

The main aim of the diagnostic course is compact presentation of the functional diagnostic methods in an otorhinolaryngology department as well as the practical and varied organization of the training block.

**Materials and methods:**

The first day of the five-day training block includes a “mirror course”, in which students learn how to perform an ENT mirror examination. After rotation in the wards, the outpatient department, and the operating rooms, the diagnostic course is completed on day 5, in which four different stations comprising practical ENT diagnostic exercises are passed through.

**Results:**

The evaluation of the practical training block showed a significant improvement after introduction of the diagnostic course: in the 2019 summer semester, 38% of *n* = 105 had grade 1.4. Personal feedback and requests for clinical traineeships also underlined this positive assessment.

**Conclusion:**

The advantages of our diagnostic course are the opportunity to apply practical skills and the small group sizes (maximum five students). The discipline of otorhinolaryngology and its varied character could be optimally presented in this manner. Disadvantageous are the high personnel and logistic requirements, which should, however, be met during university teaching.

Fehlende praktische Übungen sowie fehlende klinische Relevanz im Rahmen der Praktika im klinischen Abschnitt des Humanmedizinstudiums sind ein häufiger Kritikpunkt in den Evaluationen der Studierenden. Dies betrifft auch die Lehre einer Klinik für Hals-Nasen-Ohren(HNO)-Heilkunde. Wir etablierten einen Diagnostik-Parcours zur Darstellung der Sinne unseres Faches, um so die Lehre für unsere Studierenden anschaulicher, praktischer und abwechslungsreicher zu gestalten.

## Hintergrund

Durch die Reform der Approbationsordnung im Jahr 2002 wurden neue Herausforderungen an die Lehre im Medizinstudium gestellt [5]. Leitgedanken des Gesetzgebers für die Approbationsordnung sind die Verbesserung der Ausbildung hin zu mehr Praxisnähe [3].

Es ist dabei selbstverständlich, dass das Üben von praktischen Fähigkeiten einen großen Stellenwert einnehmen muss [6]. Diverse Studien haben außerdem die Bedeutung von praktischen Übungen in diagnostischen und therapeutischen Interventionen gezeigt [4]. Darüber hinaus erfordert die moderne Zeit immer mehr innovative und integrierte Lernformen [5]. Im Folgenden stellen wir eine neue Lernform zur Ergänzung des Blockpraktikums der Hals-Nasen-Ohren-Heilkunde vor.

An der Fakultät für Medizin der Universität Regensburg besteht die Lehre des Lehrstuhls für Hals-Nasen-Ohren-Heilkunde aus einer fünftägigen Vorlesung mit jeweils vier Stunden Dauer sowie einem einwöchigen Blockpraktikum. Beides findet im sechsten klinischen Semester statt. Im vergangenen Wintersemester 2019/2020 wurden 114 Studierende in der Lehre der Klinik für Hals-Nasen-Ohren-Heilkunde betreut.

**Figure 1:**
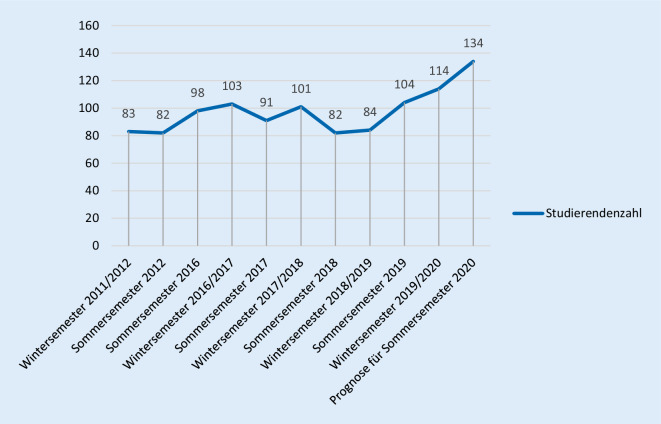

Ausschlaggebend für die Umstrukturierung des Blockpraktikums der HNO-Heilkunde waren die steigenden Studierendenzahlen in den vergangenen Semestern. So mussten im Wintersemester 2011/2012 lediglich 83 Studierende und im Wintersemester 2016/2017 bereits 103 Studierende unterrichtet werden, dieser Anstieg ist in Abb. 1 dargestellt. Die teilnehmenden Studierenden am Blockpraktikum der Klinik für Hals-Nasen-Ohren-Heilkunde decken sich aufgrund der curricularen Struktur des Humanmedizinstudiums in Regensburg mit den Studierendenzahlen der einzelnen Semester.

Ziel des neu etablierten Praktikums war u. a. eine maximale Gruppengröße von 5 Studierenden. Aufgrund der Semesterstruktur des sechsten Semesters erstreckt sich das Blockpraktikum über 8 Wochen, in denen jeweils 4 Gruppen betreut werden können. Somit ergibt sich eine maximal zu unterrichtende Studierendenzahl von 160 (8 [Wochen] *4 [Gruppen] *5 [Studierende]) pro Semester.

## Ziel

Das vorwiegende Ziel der Einführung des Diagnostik-Parcours war die kompakte Darstellung der Funktionsdiagnostik in einer universitären HNO-Abteilung als auch gleichzeitig die praktische und abwechslungsreiche Gestaltung sowie die Weiterentwicklung des bestehenden Blockpraktikums.

## Methoden

Am ersten Tag des fünftägigen Blockpraktikums wird ein „Spiegelkurs“ durchgeführt, bei dem die Studierenden lernen, einen HNO-Spiegelbefund zu erheben. An drei weiteren Tagen erfolgt die Einteilung auf einer der beiden HNO-Stationen, in der Poliklinik sowie in den Operationssälen der HNO-Abteilung. Am 5. Tag erfolgt zunächst ein zweistündiges Seminar der Abteilung für Phoniatrie und Pädaudiologie, danach wird der im Folgenden im Detail beschriebene Diagnostik-Parcours durchgeführt.

## Übersicht

Das Blockpraktikum wird über acht Wochen abgehalten, sodass bei einer durchschnittlichen Anzahl pro Semester von aktuell 100–120 Studierenden die Gruppengröße pro Woche bei etwa 12–14 Studierenden liegt. In jeder Woche wird im Vorfeld ein Rotationsplan erstellt, sodass die Studierenden in vier Gruppen von etwa drei bis fünf Studierenden eingeteilt sind. Diese durchlaufen dann für jeweils 30 min die vier Stationen des Diagnostik-Parcours. Hierfür wurden eine Station für Halssonographie, eine Station in der Audiologie, eine Station für eine Riech- und Schmeckuntersuchung sowie eine Station für Vestibularisdiagnostik in vier verschiedenen Räumen eingerichtet. Diese sind mit dem Rotationsplan beschildert. In den einzelnen Stationen sollen die Studierenden an sich selbst bzw. gegenseitig die vorgestellten diagnostischen Maßnahmen ausprobieren. Begleitet wird dies jeweils von einem Tutor, welcher die Untersuchung anleitet und parallel theoretisches Wissen dazu vermittelt.

Nach einer kurzen Erklärung des Ablaufplans, der einzelnen Stationen sowie der Vorstellung der Tutoren werden die Studierendengruppen in ihre jeweils erste Parcours-Station gebracht.

## Stationen des Parcours

**Figure 2:**
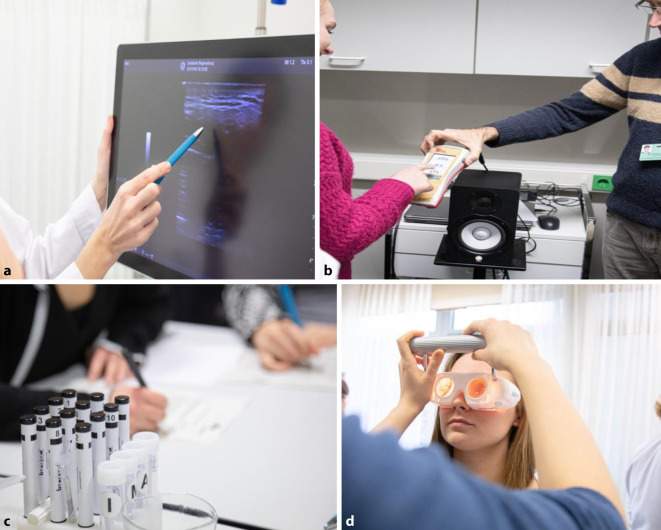

In der Station „Halssonographie“ wird es in Gruppen von bis zu 5 Studierenden jedem Studierenden ermöglicht, unter Anleitung eine sonographische Untersuchung des Halses durchzuführen. Hierzu gehört die Darstellung der Hals-Gefäß-Nerven-Scheide, der großen Speicheldrüsen, der Schilddrüse und des Mundbodens (Sonographiegerät GE Healthcare, Fa. Logiq S7 Expert, 8200 W Tower Ave, Milwaukee, WI 53223, USA), Abb. 2a).

Bei der Station „Audiologie“ lernen die Studierenden innerhalb der Gruppe, ein Tonaudiogramm zu erstellen und zu beurteilen. Weiterführende Diagnostiken wie Impedanz, sprachaudiometrische Methoden sowie objektive Messungen werden knapp erwähnt (Aurical Plus Audiometer; Abb. 2b).

In der Station „Riech- und Schmeckuntersuchung“ durchlaufen die kleinen Studierendengruppen diese beiden subjektiven Messmethoden (Riechstreifen und Schmeckstifte Sniffin’Sticks der Fa. Burghart, Tinsdaler Weg 175, 22880 Wedel, Deutschland). Gleichzeitig werden diese praktischen Übungen von einer Präsentation zur Anatomie, Physiologie und Nomenklatur der Riech- und Schmeckfunktionen begleitet (Abb. 2c).

In der Station „Vestibularisdiagnostik“ erfolgt ein halbstündiges Seminar, in dem vor allem die klinisch-praktische Abklärung eines Patienten mit akut aufgetretenem Schwindel mittels Frenzel-Brille und klinischen Tests erklärt wird. Darüber hinaus werden knapp die ergänzenden Methoden der vestibulären Diagnostik wie Kopfimpulstest und Elektronystagmographie besprochen (Abb. 2d).

Bei diesen Stationen handelt es sich um häufige diagnostische Maßnahmen einer HNO-Klinik. Sie sind außerdem gut an gesunden Studierenden gegenseitig wiederholt durchführbar, ohne dass sie gesundheitliche Schäden verursachen könnten.

**Figure 3:**
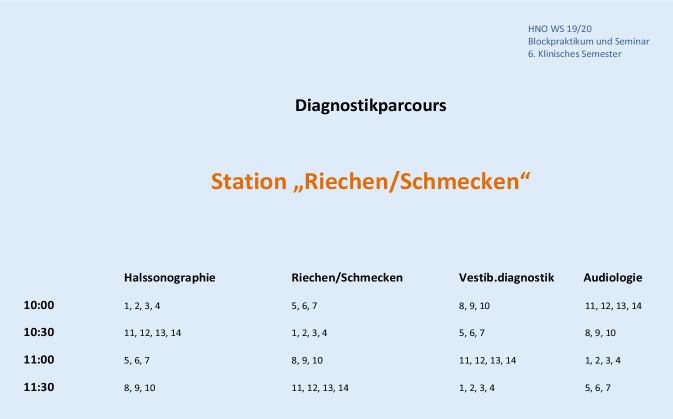

Der Diagnostik-Parcours wurde in den Räumlichkeiten der Poliklinik der Hals-Nasen-Ohren-Heilkunde (Audiologie, Sonographie‑, Funktionsdiagnostik-Raum und Konferenzraum) durchgeführt. Diese Räume wurden für die Zeit dieses Studierendenunterrichts während der acht Wochen dafür freigehalten und entsprechend beschildert (Beispiel in Abb. 3).

Die Tutoren der vier Stationen bestehen aus zwei ärztlichen Mitarbeitern (Halssonographie, Vestibularisdiagnostik), einem/r Mitarbeiter/in der Audiologie sowie einer medizinisch-technischen Angestellten (Riech- und Schmeckuntersuchung). Da das Blockpraktikum eine Anwesenheitspflicht erforderlich macht, erfolgt die Bestätigung der Anwesenheit am Ende des Diagnostik-Parcours.

## Ergebnisse

Vor Einführung des Diagnostik-Parcours wurde das Praktikum der HNO-Heilkunde am Universitätsklinikum Regensburg (UKR) in den zwei vorangegangenen Evaluationen mit der Note 1,7 bewertet (Wintersemester 2017/2018, 58 % von *n* = 101, Standardabweichung s = 0,7; Wintersemester 2018/2019, 35 % von *n* = 84, s = 0,9). Die durchschnittliche Note der Gesamtbewertung des Praktikums über die evaluierten Semester vom Sommersemester 2012 bis inklusive des Wintersemesters 2018/2019 betrug 1,83 (*n* = 389, s = 0,79).

Trotz dieser konstant guten Bewertung und nach Auswertung der Freitext-Evaluationen erschien uns noch Optimierungspotenzial vorhanden zu sein. Vor allem bezog sich die Kritik meist auf eine zu langatmige und einseitige Vermittlung von spezieller Diagnostik, ohne einen Überblick über die verschiedenen Diagnostik-Einheiten zu erlangen. Die Lehre von Diagnostik am Patienten erwies sich als nicht praktikabel, da hierbei die angebotenen Fälle eher zufällig, damit gehäuft oder gar nicht bzw. wenig strukturiert auftauchten.

### Evaluation

Der von uns beschriebene Parcours wurde bis dato während zwei Semestern durchgeführt. Anschließend erfolgte die Evaluation durch die Studierenden, hierbei handelt es sich nach Kirkpatrick um eine Evaluation auf der ersten Ebene, der Reaktionsebene [2].

Es erfolgte der Vergleich der Evaluation des Sommersemesters 2019 (mit Diagnostik-Parcours) einerseits mit dem Durchschnitt der Bewertung aus den Semestern Sommersemester 2012 bis inklusive Wintersemester 2018/2019 und andererseits mit dem unmittelbar davor stattfindenden Wintersemester 2018/2019. Zur Errechnung der Signifikanz wurde der Welch-t-Test angewandt. Als signifikant wurden Ergebnisse mit einem *p* < 0,05 (*) gewertet.

Wir konnten unsere Evaluation des Blockpraktikums im Gegensatz zum Durchschnitt der vorhergehenden Jahre signifikant verbessern, im Vergleich zum direkt vorangegangenen Semester ergaben sich keine signifikanten Verbesserungen (Sommersemester 2019: Note 1,4 bei 38 % von *n* = 105, s = 0,9; vs. Wintersemester 2018/2019: Note: 1,7, 35 % von *n* = 84, s = 0,9, *p* = 0,173; und vs. Durchschnitt Sommersemester 2012 bis inkl. Wintersemester 2018/2019, Note 1,83, *n* = 389, s = 0,79, *p* = 0,006**).

**Figure 4:**
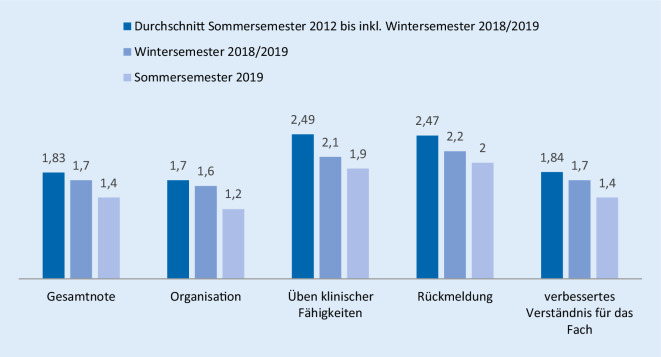

Insbesondere die Evaluationsergebnisse für die Bereiche „Organisation“ (Durchschnitt Sommersemester 2012 bis inkl. Wintersemester 2018/2019, Note 1,7, s = 0,91, *p* = 0,002**; und Wintersemester 2018/2019, Note 1,6, s = 1,2, *p* = 0,132; vs. Sommersemester 2019, Note 1,2, s = 0,9), „Üben klinischer Fertigkeiten“ (Durchschnitt Sommersemester 2012 bis inkl. Wintersemester 2018/2019, Note 2,49, s = 1,26, *p* = 0,009**; und Wintersemester 2018/2019, Note 2,1, s = 1,0, *p* = 0,470; vs. Sommersemester 2019, Note 1,9, s = 1,3), „Rückmeldung“ (Durchschnitt Sommersemester 2012 bis inkl. Wintersemester 2018/2019, Note 2,47, s = 1,27, *p* = 0,023*; und Wintersemester 2018/2019, Note 2,2, s = 1,0, *p* = 0,450; vs. Sommersemester 2019, Note 2,0, s = 1,2) und „verbessertes Verständnis für das Fach“ (Durchschnitt Sommersemester 2012 bis inkl. Wintersemester 2018/2019, Note 1,84, s = 0,97, *p* = 0,011*; und Wintersemester 2018/2019, Note 1,7, s = 1,1, *p* = 0,245; vs. Sommersemester 2019, Note 1,4, s = 1,0) besserten sich merklich. Diese Ergebnisse sind in Abb. 4 dargestellt.

Auch das persönliche Feedback der Studierenden zeigte, dass diese Neuerungen positiv aufgenommen wurden. Insbesondere die Möglichkeiten der praktischen und selbstständigen Übungen wurden hervorgehoben.

### Prüfungsergebnisse

Die Klausur bezieht sich auf die Inhalte der Hauptvorlesung und des Blockpraktikums und besteht aus Multiple-Choice- und offenen Fragen. Seit Einführung des Diagnostik-Parcours wurden theoretische Fragen zu diesen Inhalten ergänzt, eine praktische Erfolgskontrolle wurde bisher nicht durchgeführt. Dies wäre jedoch eine gute Alternative, um in der Zukunft die Prüfungssituation zu ergänzen.

### Nachwuchsförderung

Außerdem erhielten wir im Rahmen jedes Praktikums mehrere Anfragen – durchschnittlich von fünf Studierenden – bzgl. einer Famulatur in der HNO-Klinik. Dies zeigt das Interesse der Studierenden, welches durch ein abwechslungsreiches und praxisorientiertes Praktikum gefördert werden kann.

## Diskussion

Wesentlicher Vorteil des Diagnostik-Parcours ist die Möglichkeit, praktische Fertigkeiten direkt anzuwenden und zu üben – ein Faktor, der von Studierenden heutzutage deutlich gefordert und bei Mangel auch kritisiert wird. Entscheidend ist, dass die Qualität der Lehre u. a. von der Aufbereitung und Darstellung des Lehrinhalts abhängig ist [1].

Unsere Ergebnisse in der Evaluation zeigen, dass ein innovatives Lehrkonzept den Zugang zum Fach erleichtern und den Zugewinn an Wissen der Studierenden enorm steigern kann.

Die Auswertung der Evaluation ergab eine statistisch signifikante Besserung der Evaluation des Semesters mit Durchführung des Diagnostik-Parcours im Vergleich zum Durchschnitt aller vorhergehenden ausgewerteten Semester, jedoch nicht mit dem direkt davor liegenden Semester. Ursache hierfür könnte eine stetige Verbesserung des Lehrangebots auch in anderen Bereichen sein, sodass insgesamt die Lehre der Klinik für Hals-Nasen-Ohren-Heilkunde positiver bewertet wurde. Die praktischen Aspekte des Diagnostik-Parcours tragen jedoch sicherlich wesentlich zur Besserung der Evaluation bei. Darüber hinaus könnte die kleine Fallzahl im vorhergehenden Semester auch ein Grund für die fehlende Signifikanz bei Vergleich der beiden aufeinanderfolgenden Semester sein.

Im Fall unseres Diagnostik-Parcours ist darüber hinaus die kleine Gruppengröße von maximal fünf Studierenden, die eine individuelle und intensive Betreuung ermöglicht, als positiv zu werten. Besonders erscheint hierbei, dass Sinne, wie Hören und Riechen, in ihrer Bedeutung als fundamentaler Charakter unseres Faches hervorgehoben werden. Durch solche Neuerungen kann das Fach der Hals-Nasen-Ohren-Heilkunde mit seinem abwechslungsreichen Charakter attraktiver dargestellt werden. Vielmehr aber können praktische Fertigkeiten vermittelt werden und auch Studierende, die sich primär nicht für unser Fach begeistern, können Kenntnisse und Fähigkeiten erwerben, die auch für andere Fächer wie Pädiatrie, Allgemeinmedizin, Anästhesie oder innere Medizin essenziell sind.

Ein wesentlicher Nachteil besteht im hohen personellen Aufwand, praxisnaher Kleingruppenunterricht ist enorm Zeit- und personalintensiv [3]. Für unseren Diagnostik-Parcours müssen in dieser Zeit mindestens vier Mitarbeiter mit der Betreuung beauftragt werden. Dies sollte aber während der Semesterzeit an einer Universitätsklinik ermöglicht werden. Im Vorfeld ist die Erstellung eines Rotationsplans mit Einteilung der Studierenden, die Beschriftung der Räume sowie die Einteilung der Tutoren erforderlich.

Eine Option wäre, den Diagnostik-Parcours im Rahmen eines Peer-assisted Learning anzubieten, indem Studierende geschult werden, die dann die diagnostischen Inhalte weitergeben. Die Methodik wäre hierfür ideal geeignet, und es könnten personelle Ressourcen der Klinik eingespart werden.

In Zukunft planen wir außerdem, den Parcours um weitere diagnostische Stationen zu ergänzen, hierzu könnte z. B. eine Rhinomanometrie oder eine allergologische Diagnostik zählen.

Zusammenfassend stellt unser neu etablierter Diagnostik-Parcours eine sinnvolle und sehr positiv evaluierte Ergänzung des Blockpraktikums und damit der Lehre in der Hals-Nasen-Ohren-Heilkunde an unserer Klinik dar.

## Fazit für die Praxis

- Die diagnostischen Möglichkeiten unseres Faches können optimal in einem Diagnostik-Parcours dargestellt werden.
- Die Durchführung dieser Lehrform erscheint praktikabel und könnte für andere HNO-Kliniken ebenfalls gut umsetzbar sein.
- Qualitativ hochwertige Lehrangebote und eine gute Betreuung in Kleingruppen können durch die Verbesserung der Attraktivität der Lehre zur Nachwuchsförderung für das Fach der Hals-Nasen-Ohren-Heilkunde beitragen.
